# ω-3 Polyunsaturated Fatty Acids on Colonic Inflammation and Colon Cancer: Roles of Lipid-Metabolizing Enzymes Involved

**DOI:** 10.3390/nu12113301

**Published:** 2020-10-28

**Authors:** Maolin Tu, Weicang Wang, Guodong Zhang, Bruce D. Hammock

**Affiliations:** 1 Department of Food Science, University of Massachusetts, Amherst, MA 01002, USA; mtu@umass.edu (M.T.); guodongzhang@umass.edu (G.Z.); 2 Department of Food Science and Technology, National Engineering Research Center of Seafood, Collaborative Innovation Center of Seafood Deep Processing, Dalian Polytechnic University, Dalian 116034, China; 3 Department of Entomology and Comprehensive Cancer Center, University of California, Davis, CA 95616, USA; wcwwang@ucdavis.edu; 4 Molecular and Cellular Biology Graduate Program, University of Massachusetts, Amherst, MA 01002, USA

**Keywords:** ω-3 PUFAs, colonic inflammation, colorectal cancer, eicosanoids, cytochrome P450 monooxygenases, soluble epoxide hydrolase

## Abstract

Substantial human and animal studies support the beneficial effects of ω-3 polyunsaturated fatty acids (PUFAs) on colonic inflammation and colorectal cancer (CRC). However, there are inconsistent results, which have shown that ω-3 PUFAs have no effect or even detrimental effects, making it difficult to effectively implement ω-3 PUFAs for disease prevention. A better understanding of the molecular mechanisms for the anti-inflammatory and anticancer effects of ω-3 PUFAs will help to clarify their potential health-promoting effects, provide a scientific base for cautions for their use, and establish dietary recommendations. In this review, we summarize recent studies of ω-3 PUFAs on colonic inflammation and CRC and discuss the potential roles of ω-3 PUFA-metabolizing enzymes, notably the cytochrome P450 monooxygenases, in mediating the actions of ω-3 PUFAs.

## 1. Introduction

There are ~1.8 million new cases of and ~881,000 deaths from colorectal cancer (CRC) every year [1]. It is estimated that ~30% of cancers in developed countries are diet-related [2]. Therefore, it is important to develop effective diet-based prevention strategies to reduce CRC risks. Epidemiological and preclinical data support that ω-3 polyunsaturated fatty acids (PUFAs), such as plant-derived α-linolenic acid (ALA, 18:3ω-3) and marine fish-derived eicosapentaenoic acid (EPA, 20:5ω-3), docosapentaenoic acid (DPA, 22:5ω-3), and docosahexaenoic acid (DHA, 22:6ω-3), may reduce CRC risks, in part, through suppressing colonic inflammation. In contrast, ω-6 PUFAs, such as linoleic acid (LA, 18:2ω-6) and arachidonic acid (ARA, 20:4ω-6), are suggested to exaggerate the development of colonic inflammation and CRC [3,4,5,6,7,8]. This is important because the current Western diet has 30–50-times more ω-6 PUFAs than ω-3 PUFAs. The validation of the beneficial effects of ω-3 PUFAs on CRC will have a significant impact on public health. However, after decades of research, the anti-CRC efficacy of ω-3 PUFAs remains inconclusive, making it difficult to make dietary recommendations or guidelines of ω-3 PUFAs for CRC prevention [9]. The inconsistent results suggest that there could be more complex mechanisms, which may be subject to specific cellular and/or metabolic modulation, involved in the anticancer and anti-inflammatory effects of ω-3 PUFAs. Therefore, it is of critical importance to better understand the mechanisms behind the anticancer and anti-inflammatory activities of ω-3 PUFAs to optimize their use for CRC prevention.

A widely accepted molecular mechanism to explain the potential health-promoting effects of ω-3 PUFAs is that they can compete with ARA (a major ω-6 PUFA) for the enzymatic metabolism catalyzed by cyclooxygenase (COX), lipoxygenase (LOX), and cytochrome P450 (CYP) enzymes, leading to reduced levels of ω-6-series metabolites (termed eicosanoids) that are predominately proinflammatory and protumorigenic, and/or increased levels of ω-3-series metabolites, which have less detrimental or even beneficial effects [10,11,12,13]. A recent study showed that there is a high degree of interindividual variability in metabolizing ω-3 PUFAs to generate lipid metabolites [14]. Thus, it is feasible that polymorphisms in the genes encoding the ω-3 PUFA-metabolizing enzymes could affect the metabolism of ω-3 PUFAs, impacting the generation of bioactive lipid metabolites in tissues and contributing to observed mixed results in ω-3 PUFA studies [15]. A better understanding of the interactions of ω-3 PUFAs with their metabolizing enzymes could lead to targeted human studies to better understand the metabolic individuality and nutrition effects of ω-3 PUFAs [15,16].

In this review, we summarize recent studies of ω-3 PUFAs on CRC and colonic inflammation (inflammatory bowel disease (IBD)) and discuss the potential roles of ω-3 PUFA-metabolizing enzymes, notably the CYP enzymes, in mediating the actions of ω-3 PUFAs.

## 2. Effects of ω-3 PUFAs on CRC and IBD

### 2.1. Effects of ω-3 PUFAs on CRC

Epidemiological and preclinical studies support the preventive effects of ω-3 PUFAs on CRC. In Table 1, we focus on the recent human studies on ω-3 PUFAs, as well as previous studies that have shown the beneficial effect of the ω-3 PUFAs and have been discussed by other review articles. A meta-analysis demonstrated a small but significant ~12% reduction of CRC risk between the highest and lowest ω-3 PUFA consumption groups [17]. In the VITamins And Lifestyle (VITAL) cohort study, the individuals who routinely took fish oil supplements had lower risks of developing CRC compared with those who did not take supplements [18]. The European Prospective Investigation into Cancer and Nutrition (EPIC) study also showed that increased ω-3 PUFA consumption reduced CRC risks [19]. In a randomized, double-blind, placebo-controlled trial, EPA intake was associated with reduced polyp number and size in familial adenomatous polyposis (FAP) patients [20]. Increased intake of ω-3 PUFAs was also associated with improved disease-free survival in stage III CRC patients [21]. In a phase II double-blind, randomized, placebo-controlled trial, EPA intake increased overall survival in advanced CRC patients undergoing liver resection due to liver metastases (CRCLM) [22]. Together, these studies support the conclusion that ω-3 PUFAs reduce the risks of CRC.

Consistent with the human studies, recent animal studies also support the beneficial effects of ω-3 PUFAs on CRC (Table 2). Treatment with an ω-3 PUFA mixture or EPA reduced intestinal polyposis formation in a spontaneous intestinal cancer model (using *Apc*^Min/+^ mice) [25,26]. Dietary administration of EPA also decreased tumor incidence and multiplicity in a chemically induced colitis-associated colorectal cancer (CAC) model [27]. In addition, administration of fish oil suppressed the aberrant crypt foci number and adenoma incidence in 1,2-dimethylhydrazine (DMH) or azoxymethane (AOM)-induced CRC models in rats [28,29]. Besides dietary feeding studies using ω-3 PUFAs, previous studies also showed that *fat-1* transgenic mice, which have higher tissue levels of ω-3 PUFAs, have reduced development of CRC in both *Apc* gene mutation-induced CRC model [30] and chemically induced CAC model [31,32].

In addition to the orthotropic CRC tumor models discussed above, ω-3 PUFAs have also been shown to inhibit CRC in xenograft and metastasis models. Our recent study showed that administration of an ω-3 PUFAs-enriched diet inhibited MC38 (murine colon adenocarcinoma cell) tumor growth in a murine xenograft model [33]. Consistent with our result, fish oil- or DHA-rich diets attenuated tumor burden and aggressivity in HCT-116 or SW620 (both are human colon cancer cells) xenograft tumor models in nude mice [34,35,36]. In a MC-26 colon cancer cell-induced CRC metastasis model, treatment of EPA suppressed liver metastases in BALB/c mice [37]. In a CC531 colon cancer cell-derived liver metastasis model in rats, administration of an ω-3 PUFAs-rich diet reduced hepatic tumor incidence and burden [38]. Moreover, ω-3 PUFAs could be used to enhance the actions and reduce the toxicity of anticancer drugs. The coadministration of oxaliplatin and DHA synergistically inhibited HCT-116 xenograft tumor growth in nude mice [35]. Overall, these results support the anti-CRC effects of ω-3 PUFAs.

Human and animal studies also support that the dietary intake of ω-3 PUFAs-rich foods, such as fish, flaxseed, and walnuts, reduces the risks of CRC. In the EPIC cohort study, the consumption of ω-3 PUFAs-rich fish was linked with lower risks of developing CRC [19]. Stage III CRC patients who regularly consumed dark fish (≥1 time per week) had increased disease-free survival rates and lower cancer recurrence/motility risks compared to those who did not [21]. Consistent with the human studies, the administration of a flaxseed-rich diet reduced aberrant crypt foci formation in both proximal and distal colon in an AOM-induced CRC model in rats [39]. The intake of a walnut-added diet also attenuated tumor growth in a HT29 cell-induced CRC xenograft model in mice [40]. ω-3 PUFAs could exhibit beneficial effects via regulating microbiota during CRC. The administration of EPA increased the abundance of *Lactobacillus* in a CAC cancer model in mice [27]. The intake of EPA and DHA mixture could also increase the levels of *Bifidobacterium*, *Roseburia*, and *Lactobacillu*s in humans [41]. Though more studies are needed to determine the extent to which food components, besides the ω-3 PUFAs, contribute to the observed anti-CRC effects, these results further support the beneficial effects of ω-3 PUFAs on CRC.

Though many studies support the beneficial effects of ω-3 PUFAs on CRC, there are inconsistent results from animal and human studies. Some reports, in fact, have shown that ω-3 PUFAs had no effect [42,43] or even detrimental effects on the development of CRC [44,45] (Table 1 and Table 2). The Health Professionals Follow-Up Study (HPFS) and Nurses’ Health Study (NHS) cohort studies showed that ω-3 PUFA intake had no effect on overall CRC risks, and even increased distal colon cancer risk in certain individuals [23]. The supplementation of ω-3 PUFAs had no effect on the recurrence or survival rate in stage III colon cancer patients [46]. Moreover, in a randomized, double-blind, placebo-controlled clinical trial, compared with saline infusion, intravenous infusions of ω-3 PUFAs worsened the infectious complications in CRC patients undergoing colon resection [24]. Other postoperative complications were also reported in CRC patients who received ω-3 PUFAs after surgery [47]. Animal studies also showed that the treatment of fish oil exacerbated *Helicobacter hepaticus*-induced colitis and adenocarcinoma in SMAD3-deficient mice [45]. These inconsistent results make it difficult to effectively implement ω-3 PUFAs to reduce the risks of CRC.

### 2.2. Effects of ω-3 PUFAs on IBD

IBD, which is characterized by chronic inflammation in intestinal tissues, severely impacts the quality of life of the patients. Symptoms include abdominal pain, vomiting, diarrhea, and rectal bleeding. The incidence and prevalence of IBD have risen dramatically in recent decades: In 2015, ~1.3% of US adults (3 million) were estimated to be diagnosed with IBD [48], representing a 50% increase from 1999 (2 million) [49]. To date, there is no cure for IBD, and the current anti-IBD treatments could lead to serious side effects, including infection, bone marrow dysfunction, and organ dysfunction [50]. Therefore, it is important to develop novel preventive strategies to reduce the risks of IBD.

Human and animal studies support the beneficial effects of ω-3 PUFAs on the development of IBD. The intake of fish oil reduced the abundance and activity of cytotoxic NK cells and improved the disease condition in IBD patients [51]. Fish oil also decreased disease activity index and reduced neutrophil infiltration in ulcerative colitis (UC, a subtype of IBD) patients [52,53]. In animal models, ω-3 PUFAs suppressed T cell-transplantation-induced colitis in severe combined immunodeficient (SCID) mice [54]. The treatment of a ω-3 PUFA (using linseed oil)-rich diet reduced the incidence of ovalbumin-induced allergic diarrhea in a food allergy mouse model [55]. The intake of ω-3 PUFAs, especially the EPA, reduced tissue damage and IBD-associated diarrhea, bloody stools, and weight loss in dextran sodium sulfate (DSS)-induced colitis models in mice and rats [56,57,58]. In ischemia-reperfusion (IR) rats, the intake of ω-3 PUFA-attenuated IR-induced mucosal injury in intestine [59]. In addition to the nutritional intervention of ω-3 PUFAs, *fat-1* transgenic mice, which have higher tissue levels of ω-3 PUFAs, have been shown to exhibit reduced colonic inflammation in DSS- or 2,4,6-trinitrobenzenesulfonic acid (TNBS)-induced colitis [60,61]. ω-3 PUFAs mainly exhibit beneficial effects via regulating immune cell infiltration during IBD. The administration of ω-3 PUFAs reduced the colonic infiltration of neutrophils [53,58], macrophages [62], T cells [54], and NK cells [51] in IBD mice and patients. Moreover, ω-3 PUFAs have been shown to decrease proinflammatory cytokines (TNF-α, IL-12, IL-1β, iNOS, and/or IL-6), enhance epithelial barrier function, upregulate antioxidative enzymes, and reduce lipid oxidation-derived compounds [54,57,58,59,60,61], and therefore inhibit the development of IBD in mice or rats.

There are also inconsistent reports, which have shown that ω-3 PUFAs have no effect or even adverse effects on IBD. In randomized, placebo-controlled trials, ω-3 PUFAs intake has had no effect in improving the recovery of colitis [63,64], and has even enhanced disease activity in UC patients [65]. Moreover, ω-3 PUFAs had no effect on either chemotherapy-induced enterocolitis in acute myeloid leukemia (AML) patients [66] or type 2 diabetes-induced duodenal inflammation in obesity patient [67]. In animal models, the treatment of fish oil has had little effect on DSS- or TNBS-induced colitis in rats [68,69], and has exacerbated the DSS-induced colitis in mice [70]. ω-3 PUFAs have also been shown to exaggerate chemotherapy (5-fluorouracil)-induced small intestine damage in rats [71].

### 2.3. Potential Reasons for the Mixed Results of ω-3 PUFAs

Overall, the effects of ω-3 PUFAs on CRC and IBD are controversial, making it difficult to effectively use ω-3 PUFAs for disease prevention. There are several possible reasons for the mixed results in ω-3 PUFA studies.

1. Both CRC and IBD are highly heterogeneous diseases, and previous studies have shown that ω-3 PUFAs have varied effects on different types of diseases. The plasma level of ω-3 PUFAs was negatively associated with the risks of proximal colon cancer, but with not distal colon cancer or overall CRC risk [19]. The consumption of ω-3 PUFAs decreased the risks of developing rectal cancer but increased the risks of developing distal colon cancer in men [23]. The administration of fish oil reduced the aberrant crypt foci and adenoma incidence, but not the carcinoma incidence, in a DMH-induced CRC model in rats [28]. It is feasible that ω-3 PUFAs target some specific types of colon carcinogenesis or inflammation, which remains to be better defined.
2. Interindividual genetic variations could also influence the effects of ω-3 PUFAs on CRC and IBD. Many human studies have demonstrated significant interindividual variations in response to ω-3 PUFAs [14,15,72,73,74,75,76], which has made it difficult to confirm the efficacy of ω-3 PUFAs. The continuation of the current ω-3 PUFA research paradigms that neglect interindividual variation can be expected to keep generating mixed results and to fail to clarify their effects [15,16]. Notably, recent research supports that ω-3 PUFA-metabolizing enzymes contribute to the biological actions of ω-3 PUFAs. A recent study showed that there is a high degree of interindividual variability in metabolizing ω-3 PUFAs to generate lipid metabolites [14]. In addition, many studies support the critical roles of ω-3 lipid metabolizing enzymes in the activities of ω-3 PUFAs. For example, Dwyer et al. [75] showed that a diet rich in ω-3 PUFAs decreased, while a diet rich in ω-6 PUAFs increased, the risks of atherosclerosis in the subpopulation carrying a specific 5-LOX genotype but not in the general population. Other studies have also supported that polymorphism in genes encoding lipid-metabolizing genes affect the effects of ω-3 PUFAs on CRC. Notably, in a population-based case-control study, lower DHA consumption is linked to increased CRC risk in individuals with polymorphic variants in the PTGS1 gene [74]. The ω-3 PUFAs consumption only increased disease-free survival rate in CRC patients with upregulation of the PTGS2 gene [21,77]. These results emphasize the need to better understand the roles of lipid metabolism in the actions of ω-3 PUFAs.
3. Contamination and impurities in medication, supplements, and products can potentially compromise the protective effects of ω-3 PUFAs in clinical applications. ω-3 PUFAs are highly unstable and are easily oxidized. Oxidized ω-3 PUFAs release lipid peroxidation/oxidative products, which are cytotoxic and genotoxic to colonic cells [78,79]. Moreover, persistent organic pollutants (POPs) and foreign contaminations in fish oil supplements could exacerbate the colon carcinogenesis by stimulating aberrant crypt foci formation in rats [80]. The use of high-quality ω-3 PUFAs is critical in future human and animal studies to exclude the potential adverse effects from lipid oxidative products and contaminations. In addition, multiple studies have shown that the beneficial effects of ω-3 PUFAs, including anti-inflammation [81,82], anti-atherosclerosis [83], and anti-metastasis [84] effects, are dose-dependent. More studies are needed to determine the optimal dose and treatment time to maximize the beneficial effect of ω-3 PUFAs and to establish the official recommended daily intake for the general public and for CRC patients.

## 3. Roles of ω-3 PUFA Metabolism in Mediating Inflammation and Cancer

### 3.1. Enzymatic Metabolism of ω-3 PUFAs

A widely accepted hypothesis explaining the effects of ω-3 PUFAs is that they can compete with ARA for the enzymatic metabolism catalyzed by COX, LOX, and CYP enzymes, leading to reduced levels of ω-6-series eicosanoids that are predominately proinflammatory and proangiogenic, and/or increased levels of ω-3-series metabolites, which have less detrimental or even beneficial effects [10,11,12,13]. For example, EPA can effectively compete with ARA for metabolism by cyclooxygenase-2 (COX-2), leading to reduced tissue concentrations of ARA-derived prostaglandin E_2_ (PGE_2_) which has potent proinflammatory and protumorigenic actions, and increased concentrations of EPA-derived prostaglandin E_3_ (PGE_3_), which is less proinflammatory [13,85]. The 5-lipoxygenase (5-LOX) metabolite of DHA, 4-hydroxy-docosahexaenoic acid (4-HDHA), has a potent antiangiogenic effect, while the corresponding metabolite from ARA stimulates angiogenesis [12]. Another DHA metabolite, 17-hydroxy-docosahexaenoic acid (17-HDHA), which is produced by the actions of 15-LOX, has shown anti-inflammatory in experimental colitis and arthritis models [86].

**Figure 1:**
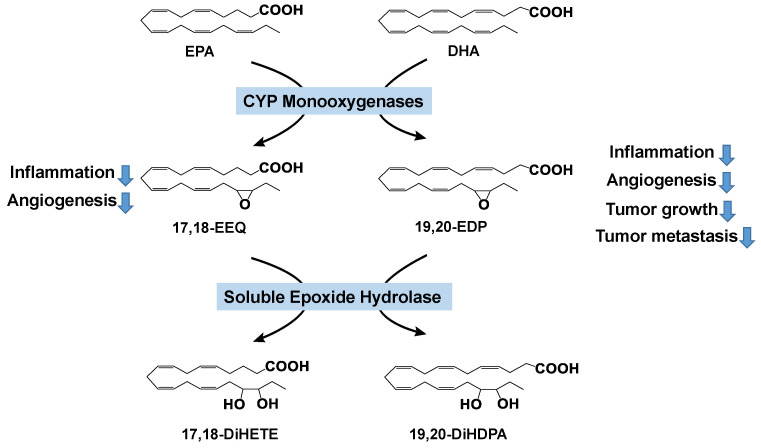
Cytochrome P450 (CYP) pathway-mediated metabolism of ω-3 PUFAs. EPA, eicosapentaenoic acid; DHA, docosahexaenoic acid; EEQ, epoxyeicosatetraenoic acid; EDP, epoxydocosapentaenoic acid; DiHETE, dihydroxy-eicoxatetraeonic acid; DiHDPA, dihydroxy-docosapentaenoic acid.

Whereas previous mechanistic research of ω-3 PUFAs has focused on the COX and LOX pathways [12,13,85,87,88,89,90,91,92,93], the role of the CYP pathway, which is widely regarded as the third branch of the eicosanoid cascade, in the metabolism and activity of ω-3 PUFAs is understudied. CYP monooxygenases (mainly CYP2C and CYP2J isoforms) convert PUFAs to the corresponding mono-epoxides (Figure 1). A series of PUFAs, including ω-6 PUFAs (LA and ARA) and ω-3 PUFAs (EPA and DHA), are substrates of the CYP enzymes. For example, DHA has six C=C double bonds, and can be converted by the CYP enzymes to generate six regioisomers: 4,5-, 7,8-, 10,11-, 13,14-, 16,17-, and 19,20-epoxydocosapentaenoic acid (EDP). Previous studies support that in many tissues, 19,20-EDP is the most abundant isomer [94,95]. Recent research by us and others has shown that ω-3 PUFAs are mainly metabolized by the CYP pathway [94,96,97,98,99], and they are known to be poor substrates of other lipid metabolizing enzymes, such as COX and LOX [100,101,102].

### 3.2. Effects of CYP-Produced ω-3 PUFA Metabolites on Inflammation and Cancer

Recent research by us and others has shown that EDPs, which are metabolites of DHA produced by CYP monooxygenases, potently inhibited angiogenesis, tumor growth, and metastasis, both in vitro and in vivo [103,104]. We showed that systematic treatment of 19,20-EDP (dose = 0.05 mg/kg/day), which was stabilized by the coadministration of a soluble epoxide hydrolase (sEH) inhibitor, inhibited tumor growth in a Met-1 breast tumor model in FVB mice and attenuated lung tumor metastasis in a Lewis lung carcinoma (LLC)-derived lung metastasis model in C57BL/6 mice [103]. Our recent study also showed that treatment with EDPs (dose = 0.5 mg/kg/day) reduced primary tumor growth in a MC38 xenograft CRC model in mice [33]. The anticancer effects of EDPs could be due to their anti-angiogenic actions. EDPs, including 7,8-, 10,11-, 13,14-, 16,17-, and 19,20-EDP, potently inhibited vascular endothelial growth factor (VEGF)-induced angiogenesis, as assessed by a Matrigel plug assay in mice [103]. EDP also inhibited VEGF-induced cell migration and tube formation in cultured endothelial cells [103]. Treatment with 19,20-EDP, or dietary feeding of ω-3 PUFAs, in Tie2-CYP2C8-Tr mice and sEH (soluble epoxide hydrolase) deficiency mice, suppressed pathological angiogenesis in a laser-induced choroidal neovascularization model [104,105].

Epoxyeicosatetraenoic acids (EEQs), which are metabolites of EPA produced by CYP monooxygenases, have been shown to have potent anti-inflammatory effects. Treatment of 17,18-EEQ decreased the incidence of ovalbumin-induced allergic diarrhea, as well as the severity of cholera toxin-induced cholera diarrhea, in murine intestinal diarrhea models [55]. 17,18-EEQ also reduced 2,4-dinitrofluorobenzene-induced contact hypersensitivity in both murine and cynomolgus macaques models of contact dermatitis [106]. Combined treatment of 17,18-EEQ and HEPEs (5-HEPE and 9-HEPE) reduced macrophage migration in adipose tissue in HFD-induced obese mice [107]. Treatment of 17,18-EEQ attenuated pathological angiogenesis in a laser-induced CNV model [104].

In addition to animal data, ex vivo and in vitro data also support the anti-inflammatory actions of EEQ. 17,18-EEQ alleviated methacholine-triggered Ca^2+^ hypersensitivity and its associated hyperresponsiveness in an ex vivo bronchial inflammatory model [108]. 17,18-EEQ reduced methacholine-induced contractile responses in a guinea pig airway explant [109]. The treatment of mixed EEQ isomers or 17,18-EEQ suppressed the activation of the JNK signaling pathway and attenuated inflammatory responses in palmitate-triggered macrophage cells [107]. Together, these results support the anti-inflammatory and antiangiogenic effects of EEQ.

17,18-EEQ can be further metabolized by 12-LOX to form 12-hydroxy-17,18-epoxyeicosatetraenoic acid (12-OH-17,18-EEQ). Both 17,18-EEQ and 12-OH-17,18-EEQ inhibited zymosan-induced peritonitis by limiting neutrophil infiltration in peritoneal lavages in a murine peritonitis model [110]. The administration of 12-OH-17,18-EEQ, instead of 17,18-EEQ, reduced airway inflammation in an ovalbumin-induced asthma model [111]. 12-OH-17,18-EEQ has also been shown to attenuate LTB_4_-induced neutrophil mobility and activation [110]. Together, these results support that EEQs and its downstream metabolites have anti-inflammatory effects.

Opposite to the effects of ω-3 metabolites (EDPs and EEQs), the corresponding ω-6 metabolites, such as LA-derived epoxyoctadecenoic acids (EpOMEs) and ARA-derived EETs, have been shown to enhance tumorigenesis [112,113]. Notably, our recent research showed that systematic treatment with 12,13-EpOME increased tumor multiplicity and tumor size in an AOM/DSS-induced CRC model in mice, demonstrating its CRC-enhancing effects [112]. We further showed that treatment with EpOME, at nM concentrations, induced inflammatory responses in macrophage cells and colon cancer cells [112]. Overall, these results suggest that ω-3 vs. ω-6 metabolites produced by the CYP enzymes have opposite effects on CRC, supporting the hypothesis that the CYP pathway could contribute to the anti-CRC effects of ω-3 PUFAs.

### 3.3. Roles of CYP Pathway in the Pathogenesis of CRC

Our recent research showed that the CYP pathway is upregulated in CRC and contributes to the pathogenesis of CRC [112]. Compared with healthy control mice, the concentrations of CYP-produced lipid metabolites are increased in the colon and plasma of AOM/DSS-induced CRC mice. The expression of a series of mouse Cyp genes, including *Cyp2c38*, *Cyp2c39*, *Cyp2c55*, *Cyp2c65*, *Cyp2c70*, *Cyp2j6, Cyp2j9*, and *Cyp2j13*, were increased in the colon tumor tissue of AOM/DSS-induced CRC mice [112]. Pharmacological inhibition or genetic ablation of CYP monooxygenases attenuated the development of AOM/DSS-induced CRC in mice, supporting the conclusion that the CYP enzymes play critical roles in the pathogenesis of CRC. In agreement with our finding, previous studies have also shown that, compared with matched benign samples, CYP2C9 is upregulated in human colon tumor samples [114]. Overall, these results support that the previously unappreciated CYP pathway contributes to colon tumorigenesis.

SEH is a downstream enzyme of the CYP-mediated lipid metabolism pathway (Figure 1). It converts the fatty acid epoxides to the corresponding fatty acid diols [115]. Recent research supports that the sEH enzyme also plays critical roles in colonic inflammation and CRC. SEH is upregulated in colonic dysplasia and adenocarcinoma samples from UC or CRC patients [114,116], and is also upregulated in colon tissues in obesity-induced colonic inflammation and food allergen-trigged intestinal inflammation models [117,118,119]. Inhibition of sEH has been shown to stabilize fatty acid epoxides and enforce their anti-inflammation effects in colitis and CRC. Inhibition of sEH reduced the ulcer formation by increasing the fatty acid epoxide levels, decreasing colonic cytokine (*Tnf-α*, *Il1β*, *Mcp-1*) expression and neutrophil infiltration in the *Il10^−/−^* and DSS-induced colitis models [116,120,121]. Moreover, sEH deficiency reduced tumor incidence and precancerous dysplasia in an *Il10^−/−^*-associated cancer model and CAC cancer model [116,122]. Our recent study showed that the inhibition or ablation of sEH attenuated obesity-induced colonic inflammation, gut leakage, activation of the protumorigenic Wnt pathway, and systemic inflammation [117,118]. Together, these studies suggest that sEH also contributes to the pathogenesis of colonic inflammation and CRC.

### 3.4. Therapeutic Benefit of sEH inhibitors with ω-3 PUFAs Combination in Inflammation and Cancer

Recent research supports that coadministration of sEH inhibitors synergizes with ω-3 PUFAs to suppress disease development. The administration of sEH inhibitor t-TUCB attenuated obesity-induced hepatic steatosis in *fat-1* mice [123]. Treatment with sEH inhibitors in *fat-1* mice elevated ω-3 series fatty acid epoxides (17,18-EEQ and 19,20-EDP) in liver and facilitated macrophage polarization from M1 into M2 phenotypes [123]. Moreover, the combined administration of 17,18-EEQ and sEH inhibitor AUDA attenuated methacholine-induced hyperresponsiveness and TNF-α-induced calcium hypersensitivity in an ex vivo bronchial inflammatory model [108].

In cancer treatment, the combined administration of 19,20-EDP and sEH inhibitor suppressed Met-1 cell-derived breast tumor growth in FVB female mice [103]. Interestingly, regorafenib, a widely used anticancer drug, is a potent inhibitor of sEH [124]. Acting as sEH inhibitor, regorafenib administration increased plasma levels of 11,12-EET, 14,15-EET, and 19,20-EDP in plasma in liver cancer patients, which could facilitate the synergistic action of DHA and regorafenib in cancer treatment [125]. In a mouse xenograft tumor model of renal carcinoma, the combined treatment of DHA and regorafenib results in greater inhibition in tumor growth and invasiveness compared to individual treatment [126]. Moreover, multiple orally active sEH inhibitors are available commercially. Two potent inhibitors have undergone multiple human safety trials, and one is entering phase 1b of human trials [127,128]. Together, the combination of sEH inhibition and ω-3 PUFAs demonstrate attractive and promising therapeutic applications in treating inflammation-related diseases. More studies are needed to explore the potential synergistic effect of ω-3 PUFAs and sEH inhibitors in colonic inflammation and CRC.

## 4. Summary

ω-3 PUFAs have been widely accepted as dietary supplements and prescription agents used in the US. Accumulating evidence from epidemiologic, clinical, and preclinical studies demonstrate the beneficial actions of ω-3 PUFAs in combatting CRC and its protective effects in attenuating IBD in humans and animals. However, studies have consistently questioned the efficacy of ω-3 PUFAs, especially in clinical application, due to their contradictory effects. The conflicting results are mainly due to the complexity of the disease, insufficient population number, inappropriate placebo selection, and interindividual genetic and metabolism variance. Further studies are warranted to clarify the underlying reasons leading to the inconsistent results and explore the unknown mechanism of beneficial action of ω-3 PUFAs.

The ω-3 PUFAs act mainly via the formation of bioactive lipid metabolites, which have potent effects to regulate inflammation and homeostasis [10,11,12,103,129]. However, the specific lipid metabolizing enzymes and lipid metabolites involved in the anticancer activities of ω-3 PUAFs are largely unknown. The elucidation of the specific lipid-metabolizing pathways and metabolites required for the anti-inflammatory and anticancer effects of ω-3 PUFAs will greatly facilitate the development of ω-3 PUFA biomarkers, leading to optimized use of ω-3 PUFAs for cancer prevention. Recent studies by us and others support that the CYP monooxygenase pathway plays a critical role in the pathogenesis of CRC [112]. In addition, the CYP-produced ω-3 metabolites (e.g., EDPs from DHA) inhibited angiogenesis, tumor growth, and tumor metastasis [103], while the CYP-produced ω-6 metabolites (e.g., EETs from ARA and EpOMEs from LA) increased tumorigenesis [112,113]. These results support the hypothesis that the previously unappreciated CYP pathway could contribute to the anticancer effects of ω-3 PUAFs. Further studies are needed to test this hypothesis, which can help to elucidate the molecular mechanisms and clarify the health-promoting effects of ω-3 PUFAs, as well as develop personalized nutrition strategies. Moreover, due to the complexity and interindividual variance, the use of an individual nutrient is unlikely to effectively achieve beneficial effects in clinic application, as the synergistic effect of multiple approaches in disease treatment has been proposed [130]. Given sEH as a novel therapeutic target in the pathogenesis of colonic inflammation and CRC, it is important to develop novel combined treatment of ω-3 PUFAs with sEH pharmacological inhibitors to suppress colonic inflammation and CRC.

## Figures and Tables

**Table 1:** Recent epidemiological and clinical studies of ω-3 polyunsaturated fatty acid (PUFA) supplementation for the prevention/treatment of colorectal cancer (CRC).

Study	Individuals	*N*	ω-3 PUFA Treatment	Dose	Duration	Control Treatment	Results	Reference
VITAL prospective cohort	US adults	68,109	Fish oil supplements	N/A	4+days/week for 3+years	no use	↓ CRC risk	Kantor et al., 2014 [18]
EPIC prospective cohort	European adults	521,324	Highest ω-3 PUFAs intake	>470 mg/day	Median 14.9 years	lowest ω-3 PUFAs intake	↓ CRC risk	Aglago et al., 2020 [19]
Randomized, double-blind, placebo-controlled trial	FAP patients	EPA-FFA (*n* = 28)	EPA-FFA	2 g/day	6 months	Placebo (*n* = 27)	↓ polyp diameters	West et al., 2010 [20]
CALGB adjuvant chemotherapy trial	stage III colon cancer patients	1011	Highest marine ω-3 PUFAs intake	0.33-0.57 g/day	>8 years	lowest marine ω-3 PUFAs intake	↑ disease-free survival	Blarigan et al., 2018 [21]
Double-blind, randomised, placebo-controlled trial	CRCLM patients	EPA-FFA (*n* = 43)	EPA-FFA	2 g/day	12–65 days	Placebo (*n* = 45)	↑ overall survival; no effect in disease burden and early CRC recurrence rates	Cockbain et al., 2014 [22]
HPFS and NHS cohort	US adults	123,529	Highest marine ω-3 PUFAs intake	≥ 0.30 g/d (women) ≥ 0.41 g/d (men)	24–26 years	lowest marine ω-3 PUFAs intake	No effect on overall CRC risk; ↑ distal colon cancer risk in men and women; ↓ rectal cancer risk in men	Song et al., 2014 [23]
Randomized, double-blind, placebo-controlled clinical trial	colon cancer patients	ω-3 PUFA (*n* = 21)	ω-3 PUFA intravenous infusion	0.2 g/ kg/day	night before and morning after resection surgery	Saline infusions (*n* = 23)	↑ infectious complications	Bakker et al., 2020 [24]

Abbreviations: VITAL, VITamins And Lifestyle; EPIC, European Prospective Investigation into Cancer and Nutrition; EPA, eicosapentaenoic acid; FFA, free fatty acid; FAP, familial adenomatous polyposis; CALGB, Cancer and Leukemia Group B; CRCLM, colorectal cancer liver metastases; HPFS, Health Professionals Follow-Up Study; NHS, Nurses’ Health Study; ↑, Increase; ↓, Decrease.

**Table 2:** Preclinical studies of ω-3 PUFA supplementation for the prevention/treatment of CRC.

Model	Species	ω-3 PUFA Treatment	Dose	Duration	Control Treatment	Results	Reference
*Apc*^Min/+^ mouse	C57BL/6 mouse	Fish oil	12% in diet	10 weeks	Standard diet with soybean oil	↓ intestinal polyp growth	Notarnicola et al., 2017 [25]
*Apc*^Min/+^ mouse	C57BL/6 mouse	EPA-FFA	2.5% or 5% in diet	12 weeks	AIN-93G diet with soybean oil	↓ polyp number and load in both small intestine and colon.	Fini et al., 2010 [26]
*Apc*^Min/+^ mouse	C57BL/6 mouse	Endogenous ω-3 PUFA synthesis by transgene of *fat-1*		20 weeks	*Apc*^Min/+^ mice on standard diet with safflower oil	↓ intestinal polyposis	Han et al., 2016 [30]
AOM/DSS-induced CRC model	C57BL/6 mouse	Endogenous ω-3 PUFA synthesis by transgene of *fat-1*		16 weeks	Wild-type mice on standard diet	↓ Tumor number	Han et al., 2016 [31]
AOM/DSS-induced CRC model	C57BL/6 mouse	Endogenous ω-3 PUFA synthesis by transgene of *fat-1*		11 weeks	Wild-type mice on AIN-93G diet with safflower oil	↓ incidence and growth rate	Nowak et al., 2007 [32]
AOM/DSS-induced CRC model	C57BL/6 mouse	EPA-FFA	1% in diet	15 weeks	AIN-93G diet with corn oil	↓ tumor multiplicity, incidence and maximum tumor size	Piazzi et al., 2014 [27]
DMH-induced CRC model	Wistar rat	Fish oil	18% in diet	36 weeks	AIN-93G diet with soybean oil	↓ number of aberrant crypt foci; ↓ incidence of adenoma	Moreira et al., 2009 [28]
AOM-induced CRC model	F344 rat	Fish oil	10% in diet	26 weeks	AIN-93G diet with mixed lipids	↓ colon tumor incidence and multiplicity	Reddy et al., 2005 [29]
MC38 cell-based xenograft model	C57BL/6 mouse	DHASCO Algae oil	8% in diet	5 weeks	AIN-93G diet with corn oil	↓ tumor volume and weight	Wang et al., 2016 [33]
SW620 cell-based xenograft model	Nude mouse	Fish oil	12% by calorie	6 weeks	Standard diet	↓ tumor growth and less aggressive	Bathen et al., 2008 [34]
HCT116 cell-based xenograft model	Nude mouse	DHA	10 mg/kg	every other day for 13 days	Ethanol	↓ tumor size	Jeong et al., 2019 [35]
HCT116 cell-based xenograft model	Nude mouse	DHA	3% in diet	14 days	Standard diet with sunflower oil	↓ tumor growth	Fluckiger et al., 2016 [36]
*H. hepaticus*-induced CRC model	SMAD3 deficiency mouse	Fish oil	6% in diet	12 weeks	AIN-93G diet with corn oil	↑ adenocarcinoma formation	Woodworth et al., 2010 [45]

Abbreviations: AIN, American Institute of Nutrition; AOM, azoxymethane; DSS, dextran sodium sulfate; DMH, 1,2-Dimethylhydrazine; i.p. intraperitoneal; SMAD3, mothers against decapentaplegic homolog 3; ↑, Increase; ↓, Decrease.
